# A geographical database of Iberian peatland and swob records

**DOI:** 10.1016/j.dib.2025.111971

**Published:** 2025-08-11

**Authors:** Raquel Fernandes, Miguel Geraldes, Guaduneth Chico, César Capinha

**Affiliations:** a Centre of Geographical Studies, Institute of Geography and Spatial Planning, University of Lisbon, Rua Branca Edmée Marques, 1600-276 Lisboa, Portugal; b Greifswald Mire Centre, 17487 Greifswald, Germany; c National Parks and Wildlife Service, 90 North Kings Street, Dublin, Ireland; d Laboratório Associado Terra, Instituto Superior de Agronomia, Tapada da Ajuda, 1349-017 Lisboa, Portugal

**Keywords:** Carbon sinks, Field survey, Literature review, Portugal, Spain, Wetlands

## Abstract

Recent efforts have compiled distribution data on peatlands in the Iberian Peninsula. However, the criteria used to define these ecosystems have often been derived from regions where climates are wetter and peatlands are more widespread. As a result, in this region, many peat-accumulating wetlands were overlooked. Identifying and improving the mapping of peatland distribution is crucial for their conservation and management, as they provide important ecosystem services. Therefore, we developed an updated geographical dataset of peatlands and swobs (wetlands with the potential to form peat) of the Iberian Peninsula. Records of peatlands and swob areas in Portugal and Spain were compiled through an extensive literature review. Additionally, field surveys performed between 2011-2015, 2018-2021, and 2023-2024 provided additional records. A total of 445 records were included in our dataset, representing the up-to-date knowledge on the distribution of these ecosystems in the Iberian Peninsula. The dataset allows the identification of occurrence hotspots, highlights underrepresented regions, and provides a baseline for future research and conservation planning.

Specifications Table

**Table:** 

Subject	Earth & Environmental Sciences
Specific subject area	Soil Science; Ecology.
Type of data	Type of data: Table Data format: Filtered, Processed
Data collection	Records of peatlands and swobs in Portugal and Spain (n=445) were compiled through an online literature review and field surveys. The literature review (n=398) was based on Google Scholar and Google Search, covering various knowledge fields (e.g., paleoenvironment, palynology). Field data from surveys performed by the authors (2011–2015, 2018–2021 and 2023–2024) were also included (n=47). All records were standardised and integrated into a unified dataset, and supplemented with additional information on the geographical, environmental, and socio-economic features of each site.
Data source location	Peatlands and swobs in the Iberian Peninsula. Geographical extent ranges from 36° 00’ N and 43° 47’ N latitude and 9° 29’ W and 3° 19’ E longitude. Online data collection was performed at the Centre of Geographical Studies, Institute of Geography and Spatial Planning, University of Lisbon, Rua Branca Edmée Marques, 1600-276 Lisboa, Portugal.
Data accessibility	Repository name: Geographical database of Iberian peatland and swob records Data identification number: 10.5281/zenodo.15412159 Direct URL to data: https://zenodo.org/records/15412160
Related research article	‘none’

## 1 Value of the Data

• Peatlands provide essential ecosystem services, particularly in carbon storage and sequestration. However, comprehensive distribution data for these ecosystems remain incomplete in many countries, including Portugal and Spain.
• Preventing the loss of peatland areas requires knowing their distribution. This dataset includes 445 records of peatlands and swobs across the Iberian Peninsula, significantly improving our knowledge of their distribution and showing that these ecosystems are more widespread than previously recognized.
• The dataset is a valuable resource for peatland research. By providing ground-truth records and details about their location and features, it contributes to global peatland initiatives such as the Global Peatland Assessment [1] and provides a geographical basis for local peatland management and conservation initiatives.

## 2 Background

In the Iberian Peninsula, peatlands are described mainly in the northern and north-western mountain ranges, where higher precipitation and lower temperature variability favour peat formation [2]. In the southern regions, water availability is generally lower, and peatlands are typically considered small and fragmented [3]. Nevertheless, peat deposits are observed in southern and western coastal and sublittoral wetlands, including in interdune slacks, endorheic basins, and back-swamp mires [3,4]. Although less extensive, these southern peatlands often support high floral diversity and richness [5], showing high ecological value.

Previous mapping efforts are based mostly on criteria adapted to northern peatlands, such as minimum thickness thresholds [4,6], overlooking the specificities of southern Iberian peatlands. Accordingly, effective identification and mapping of these southern areas requires adapting existing definitions and compiling region-specific data.

To address this, an extensive dataset of peatland and swob records in mainland Portugal and Spain was compiled, using region-adjusted criteria that better reflect the characteristics of Mediterranean environments [4,6,7].

## 3 Data Description

**Fig. 1:**
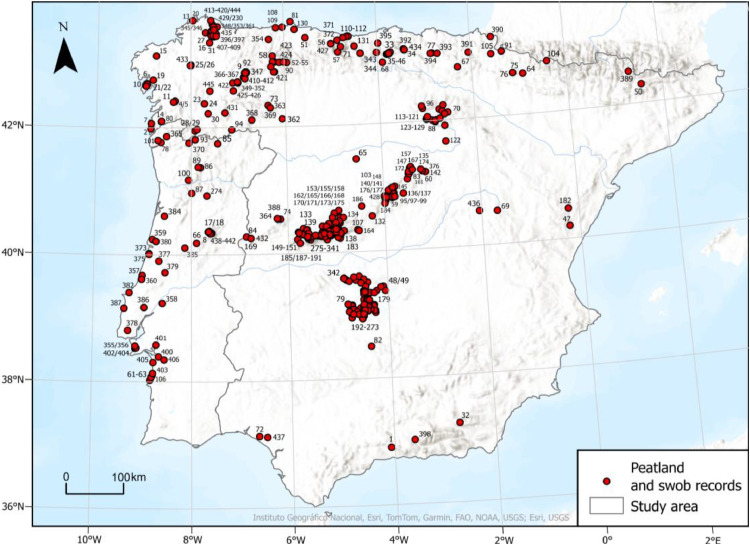
Spatial distribution of peatland and swob point records collected for Spain and Portugal, based on a literature review and field surveys.

The dataset consists of a datasheet [8] with 445 entries, each representing a geographical record of a peatland or a swob. Of these, 390 records are located in Spain and 55 in Portugal (Fig. 1). The records were compiled through an extensive literature review and supplemented by field surveys conducted by the authors.

**Fig. 2:**
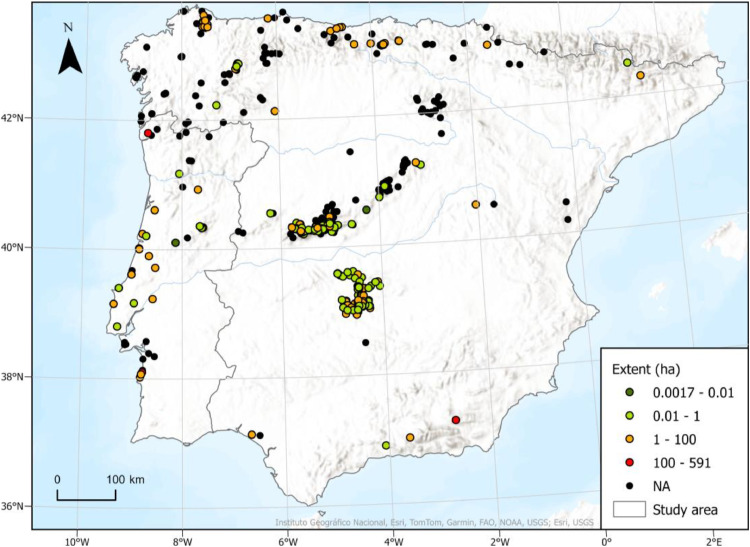
Example of a visual analysis allowed by the dataset showing the extent (ha) for each record. Black dots (NA) indicate records where this information is unavailable.

The dataset is stored in an Excel file format, named “Fernandes_et_al_Peatmap_database_v1.0.xlsx”. It contains three sheets. In the first sheet (“Description”), there is a summary of the information that can be accessed in the dataset. The second sheet, “Dataset”, contains 19 columns that identify (“ID”; “Original name”), locate (“Country”; “Municipality”; “Latitude”; “Longitude”), and characterize (“Elevation (m a.s.l.)”; “Extent (ha)”; “Characteristics from literature/fieldwork”; “Mire types based on Natura 2000 habitat”; “Maximum peat depth registered (cm)”; “Classified area/Protection Status”; “Biogeographical region”; “Mire region of Europe”; “Artificial areas (%)”; “Agricultural areas (%)”; “Forest and semi-natural areas (%)”; “Wetlands and Water Bodies (%)”) the records. The last column, “Source”, indicates the origin of each record, specifying either the literature reference from which the information was obtained or identifying it as a result of our fieldwork. The third sheet (“References”) is the list of references to the records collected in the literature review.Geographical coordinates have been standardised in decimal degrees using the standard WGS84 geographic coordinate system, facilitating the integration of location data into Geographic Information Systems (GIS) and supporting visual analysis of the spatial distribution of parameters associated with each record (e.g., Fig. 2). A “NA” code was assigned whenever it was not possible to obtain information for any of the variables.

## 4 Materials and Methods

### 4.1 Criteria

Peatland and swob records included in the dataset follow the defining criteria of Joosten *et al*. [5]. Accordingly, peatlands include any area with a naturally accumulated peat layer at the surface, even if the peat horizon does not reach the minimum 30-cm thickness threshold. Swobs correspond to any wetland with vegetation that may form peat. These criteria exclude records of buried peat and peat at the surface after erosion (i.e., former active peatland ecosystems).

### 4.2 Literature review

An initial dataset of peatland and swob records across the Iberian Peninsula was compiled through a systematic online literature review. Records were first identified and extracted from peer-reviewed peatland maps recently published for Portugal and Spain [3,4,6]. Additional records were subsequently sought from a range of complementary sources. Particular attention was given to identifying sources beyond English-language scientific literature, which was already well represented in the published maps. These included online databases, doctoral theses, books, and technical reports, potentially from different research areas (e.g., paleoenvironment, palynology, paleoecology), including documents written in Portuguese and Spanish. For that purpose, the searches were performed trough a combined use of Google Scholar and Google Search, which is considered the most comprehensive academia search engine [9].

Multiple search terms were employed, including “peat,” “peatland,” “mire,” and “swob,” along with their Spanish equivalents (“turba,” “turbera”) and Portuguese equivalents (“turfa,” “turfeira”). These terms were combined with the geographic terms “Portugal” and “Spain” to focus the search on the two countries surveyed. In addition to Spanish and Portuguese, literature in French and English was also reviewed. Only records of peatlands or swobs from documents published in 2000 or later were included in the database to ensure an up-to-date representation of peatland distribution.

### 4.3 Field surveys

**Fig. 3:**
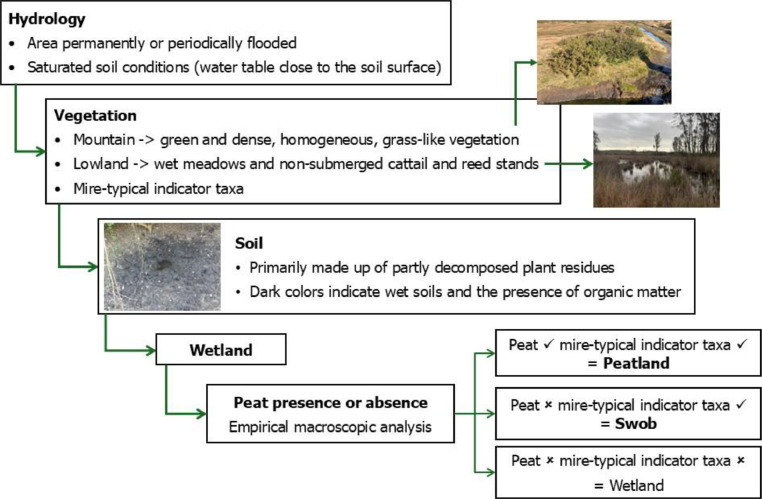
Diagram of protocol followed for identification of peatland and swob sites from field surveys. Visual identification of wetlands with potential for peatland formation followed a set of indicators related to hydrology, soil characteristics, and vegetation composition (adapted from MedWet Habitat Description System; [11]). After the visual identification of ecosystems with mire-typical indicator taxa, a macroscopic analysis of soil samples was performed to assess the presence or absence of peat. Areas with mire-typical vegetation but without peat were classified as swobs, while those containing peat were classified as peatlands.

The dataset derived from the online literature review was further supplemented with empirical data collected during field surveys performed by the authors during three main periods: 2011–2015, 2018–2021, and 2023–2024. Survey locations were selected based on the presence and richness of mire-typical indicator taxa, known to be associated with peatlands and other organic soil-forming environments across the Iberian Peninsula [10], their accessibility, and prior knowledge of local experts. The MedWet Habitat Description System protocol [11] was adapted to perform identification of wetlands with potential for peat accumulation, using essential indicators related to hydrology, soil characteristics, and vegetation composition (Fig. 3).

The presence or absence of peat was confirmed through *in situ* empirical macroscopic soil analysis, using a Royal Eijkelkamp two-piece gauge auger set with conical screw thread. This equipment allowed the accurate macroscopic assessment across a wide range of soil types, from soft peaty soils to more resistant sandy, loamy, or clayey substrates, and allowed the determination of peat depth. Four topsoil core collections were performed on each site for further analysis (soil colour and moisture, degree of humification based on von Post scale, and peat depth) based on wetter-to-drier gradient. Sites were classified based on peat empirical observation and mire-typical taxa presence or absence following Joosten *et al*. [5] (Fig. 3). Geographic coordinates for each sampling site were recorded using a handheld GPS device, allowing a spatial precision of 10m or higher.

### 4.4 Variable structure

Information for each variable in the dataset (related to the identification, location, and characterization of each record) was collected following the procedures in Table 1.

**Table 1:** Variables included in the dataset and description of the procedures and sources used to collect the corresponding data.

Group	Variable name	Description
Identification	ID	Identification number for each record, attributed by the authors.
	Original name	Retrieved from literature OR during the field surveys, based on the stream, lagoon, or locality where the record was collected.
	Country	ES = Spain; PT = Portugal
Location	Municipality	Location information was intersected with the municipality shapefile provided in the *Base de datos de divisiones administrativas de España* (2019) (Centro Nacional de Información Geográfica, 2025), for Spain, and the shapefile *Carta Administrativa Oficial de Portugal (CAOP)* [12].
	LatitudeLongitude	Retrieved from literature OR field survey measurements. UTM coordinates and degrees, minutes, and seconds were standardised into decimal degrees using the WGS84 geographic coordinate system.
	
Characterization	Elevation (metres above sea level)	Retrieved from literature OR field survey measurements, when available. If not, mean elevation was extracted from the Digital Elevation Model derived from the SRTM 1 Arc-Second Global elevation data, from the EROS Center [13].
	Extent (ha)	Retrieved from literature OR field survey measurements.
	Characteristics from literature/fieldwork	Retrieved from literature review OR field survey observation.
	Mire types based on Natura 2000 habitat	When available, Natura 2000 habitat information was retrieved from literature. If not, the records were intersected with the Natura 2000 spatial data from the European Environment Agency [14]. The habitats were classified according to Heras Pérez et al. (2017): Raised bog - 7110; Blanket bog - 7130; Fen - 7140, 7150, 7210, 7230; Para-peaty habitats (swobs) - 7140/7230.
	Maximum peat depth registered (cm)	Retrieved from literature review OR field survey measurements.
	Classified area/Protection Status	Location information was intersected with the World Database on Protected Areas (WDPA) [15].
	Biogeographical region	Retrieved from Biogeographical regions in Europe [16].
	Mire region of Europe	Retrieved from Moen et al. (2017) [17]
	Artificial areas (%)	In GIS, a 2000 m radius buffer was generated around the central coordinate of each peatland or swob record. The percentage of area covered by the CORINE Land Cover land use classes from 2018 [18], was then calculated in each buffer.
	Agricultural areas (%)	
	Forest and semi-natural areas (%)	
	Wetlands and Water Bodies (%)	
	Source	Original source of the record.

## Limitations

As with any literature-based review, the compilation of peatland records for this database is subject to inherent limitations. Not all relevant documents are available online, and some published sources lack precise geolocation data. Consequently, there is an unavoidable omission rate, and the database cannot be considered an exhaustive account of all documented peatlands in the Iberian Peninsula. Complementary fieldwork was conducted to address perceived spatial gaps. However, these surveys were also limited by site accessibility and the prevailing hydrological conditions at the time of sampling. In addition, several variables, such as peatland extent (ha) and peat depth, are only available for a subset of sites, and the geographical representation is currently limited to a single coordinate per site. Future efforts could aim to expand these attributes as new data become available and to replace point-based representations with full geographic boundaries, thereby improving spatial accuracy. Overall, this database should be seen as an updated baseline to support future mapping efforts, conservation planning, and detailed ecological assessments, while remaining open to the integration of new records and data as they become available.

## Ethics Statement

The authors have read and follow the ethical requirements for publication in Data in Brief and confirming that the current work does not involve human subjects, animal experiments, or any data collected from social media platforms.

## CRediT authorship contribution statement

**Raquel Fernandes:** Conceptualization, Data curation, Formal analysis, Investigation, Methodology, Writing – original draft, Writing – review & editing. **Miguel Geraldes:** Conceptualization, Methodology. **Guaduneth Chico:** Conceptualization, Methodology, Writing – review & editing. **César Capinha:** Conceptualization, Supervision, Writing – review & editing.

## Data Availability

ZenodoGeographical database of Iberian peatland and swob records (Original data).
